# The Role of miRNA167 in Skin Improvement: Insight from Extracellular Vesicles Derived from Rock Samphire (*Crithmum maritimum*)

**DOI:** 10.3390/biom15081157

**Published:** 2025-08-12

**Authors:** Soll Jin, ChangHoe Ku, Hye Jin Kim, Jae-Goo Kim, Sang Hoon Kim, Heyjin Han, Hee Cheol Kang, Jae Sung Hwang, Mi Jung Kim

**Affiliations:** 1 Human & Microbiome Communicating Laboratory, GFC Life Science Co., Ltd., Hwaseong 18471, Republic of Korea; s.jin@gfcos.co.kr (S.J.); hy.kim@gfcos.co.kr (H.J.K.); jg.kim@gfcos.co.kr (J.-G.K.); michael@gfcos.co.kr (H.C.K.); 2 Department of Biology, Kyung Hee University, Seoul 02447, Republic of Korea; shkim@khu.ac.kr; 3 Department of Genetics & Biotechnology, Graduate School of Biotechnology, College of Life Sciences, Kyung Hee University, Yongin 17104, Republic of Korea; changhoe.ku@y-k.co.kr; 4 GRIDA, Anseong 17577, Republic of Korea; lbtcos@naver.com

**Keywords:** extracellular vesicles, *Crithmum matrimum*, miRNA, Small RNA sequencing, miR167, skin wound healing, anti-aging

## Abstract

Samphire (*Crithmum matrimum*), a halophyte, thrives in saline environments due to its salt tolerance, which is partly attributed to miR167. However, the functional role of miR167 in human cells is unclear. This study explores the role of extracellular vesicles (EVs) derived from C. *matrimum* callus in skin regeneration, highlighting the potential of miRNA tae-miR167c-5p (miR167). Calluses were successfully induced and scaled for EV isolation. Characterization confirmed the presence of plant EV biomarkers and EVs with an average size of 136.6 nm. Cm-callus EVs enhanced wound healing and skin regeneration in human fibroblasts (HFF cells and CCD-986Sk cells) by modulating key genes, in particular, by downregulating *MMP1* and upregulating *COL1A1* and *VEGFA*. Small RNA sequencing revealed an enrichment of miR167 in Cm-callus EVs. Transfection with an miR167 mimic replicated these regenerative effects. Computational predictions identified *PPP3R2*, which is linked to the MAPK and NFAT pathways, as a potential target of miR167. This study demonstrates the efficacy of Cm-callus EVs and miR167 in promoting skin regeneration without cytotoxicity, providing insights into their therapeutic potential and calling for further experimental validation of target interactions.

## 1. Introduction

Halophytes, which have adapted to saline environments, account for only 1% of global flora. These salt-tolerant species are found in diverse saline habitats, including mudflats, sand dunes, and salt ponds [1,2]. To date, more than 2500 halophyte species have been identified [3]. Among them, rock samphire (*Crithmum maritimum*) is a representative halophyte that usually grows on cliffs and rocky shorelines and adapts to harsh environmental conditions. Rock samphire has garnered significant attention as a key active ingredient in cosmetics because of its remarkable bioactive properties that support skin health and combat aging. It has a high content of antioxidants, including phenolic compounds, vitamin C, flavonoids, carotenoids, and various phenolic acids, known for their antioxidant activity [4]. These antioxidant properties make it highly effective in preventing premature skin aging and enhancing skin resilience. Moreover, rock samphire contains vitamin B9 (folic acid) and proteins that are critical for skin regeneration and tissue-firming. These bioactive components improve skin elasticity, reduce wrinkles, and promote a youthful appearance. Because of these properties, rock samphire extracts are increasingly used in various cosmetic formulations [5].

Extracellular vesicles (EVs) are small, nanosized, lipid-bound vesicles secreted by both plant and animal cells. These vesicles encapsulate a diverse range of bioactive molecules, such as DNA, RNA, lipids, and proteins, enabling them to mediate intercellular communication by delivering their molecular cargo to target cells [6]. Most EVs have been extracted from animal cells and are divided into four categories based on their size: exosomes (50–150 nm), microvesicles (100–1000 nm), apoptotic bodies (100–5000 nm), and large oncosomes (1000–10,000 nm) [7,8]. However, researchers have recently been able to extract and characterize EVs from plant cells, which are typically 30–500 nm in size [9,10]. Plant-derived EVs (PDEVs) exhibit various therapeutic properties, including anticancer, antioxidant, and anti-inflammatory effects. Recently, PDEVs have been explored as materials for cosmetics owing to their advantages such as natural origin, safety, fewer allergic reactions, and efficient transdermal delivery [11].

MicroRNAs (miRNAs) are small single-stranded non-coding RNA molecules that typically consist of approximately 22 nucleotides. These molecules bind to the 3′ untranslated regions (UTRs) of target mRNAs to downregulate gene expression by either degrading the target mRNA or inhibiting its translation [12]. miRNAs are found in animals, some RNA viruses, and plants, where they play crucial regulatory roles. In plants, miRNAs are essential for various developmental processes, including the regulation of meristem characteristics, leaf polarity, and flowering patterns [13]. Interestingly, plant-derived miRNAs have been shown to engage in crosstalk with human cells, making them promising biomarkers for various diseases and potential components in skin care applications [14]. Among the numerous plant miRNAs, miR167 is particularly important. It plays a crucial role in the precise regulation of gene expression, contributing to the fertility of ovules and anthers and the development of roots, stems, leaves, flowers, embryos, and seeds [15,16]. Additionally, miR167 exhibits remarkable stability during storage, processing, and cooking, highlighting its tolerance to temperature changes [17,18]. Furthermore, miR167 is involved in the regulation of plant stress responses, conferring resistance to environmental challenges such as salinity, drought, and plant diseases [16,19,20].

Rock samphire, a halophyte, thrives in saline environments owing to its salt tolerance, which is partly attributed to miR167 expression. However, the functional role of miR167 in human cells remains unclear. This study aimed to explore the potential of miR167, derived from rock samphire-derived EVs in human cells, particularly in cosmetics and skin care.

## 2. Materials & Methods

### 2.1. Callus Induction of Crithmum Maritimum (C. maritimum) and Suspension Culture

For induction of callus from leaves of *C. maritimum* (Cm-callus), the leaves were soaked in 95% ethanol for 60 s and a disinfectant solution (50% bleach + 0.1% Tween-20) for 20 min. The leaf tissues were then washed at least three times with sterile water containing 500 mg/L of cefotaxime sodium (Duchefa, Haarlem, The Netherlands) as an antibiotic to remove soil pathogens. Sterilized leaves were cut into 5 × 5 mm long pieces and placed on Murashige and Skoog (MS) medium w/ Vitamins (Kisanbio, Seoul, Republic of Korea) containing 3% (*w*/*v*) sucrose and 0.35% (*w*/*v*) gelatin, along with 0.5 mg/L 2,4-dichlorophenoxyacetic acid (2,4-D, Duchefa, Haarlem, The Netherlands), 1 mg/L 6-benzylaminopurine (6-BA, Sigma-Aldrich, St. Louis, MO, USA), 1.5 mg/L 1-Naphthaleneacetic acid (NAA, Sigma-Aldrich, St. Louis, MO, USA) as callus induction medium (CIM) under dark conditions. Induced callus was subcultured 2–3 times at 4-week intervals on solid medium and cultured in suspension in the liquid medium for 3–4 weeks at a shaker speed of 100–300 rpm.

### 2.2. Isolation of Extracellular Vesicles (EVs) from Callus of C. maritimum

For isolation and analysis of EVs from Cm-callus (Cm-callus EVs), a liquid suspension culture was performed. A 4-week-cultured Cm-callus from the bioreactor was separated into callus clusters and conditioned medium through 200-mesh filters, followed by centrifugation of the medium at 4000× *g* for 20 min at 4 °C with a centrifuge to remove cell debris, fibers, and large particles. Only the supernatants were collected to eliminate cell residues, and this step was repeated to thoroughly remove impurities. The supernatant was centrifuged at 150,000× *g* for 2 h at 4 °C using an ultracentrifuge (Hitachi, Tokyo, Japan), leading to the settling of EVs in the pellet layer. The supernatants were carefully discarded without disturbing the pellet. The pellet was then resuspended in a small volume of deionized water. EVs were filtered through a 0.22 μm syringe filter (Advantec, Tokyo, Japan) for sterilization and stored at 4 °C until use. The size distribution and particle concentration of EVs were analyzed via NTA (Nanoparticle Tracking Analysis), and the zeta potential of EVs was analyzed using ZetaView (Particle Metrix, Inning, Germany). The concentration of isolated Cm-callus EVs was determined to be 2.6 × 10^10^ particles/mL. From this, the concentrations of 10% and 1% Cm-callus EVs were calculated as 2.6 × 10^9^ and 2.6 × 10^8^ particles/mL, respectively. These concentrations were used in subsequent experimental treatments to assess the biological effects of EVs at different dosages.

### 2.3. Transmission Electron Microscope (TEM) Imaging

Cm-callus EVs were placed on carbon-coated grids (EMS, Houston, TX, USA) and incubated for 5 min at room temperature (RT). Excess samples were removed, and the grids were allowed to dry. The EVs were then stained by briefly applying 2% uranyl acetate solution (EMS, Houston, TX, USA). Imaging was performed using a transmission electron microscope (JEOL, Tokyo, Japan) to visualize the morphology of EVs.

### 2.4. Western Blot Analysis

EVs were lyophilized using a freeze dryer (IlShinBioBase, Dongducheon, Republic of Korea) and lysed with 1X RIPA buffer (T&I, Seoul, Republic of Korea) on ice. Total protein extracts were separated using 10% NuPAGE^TM^ Bis-Tris Mini Protein Gels (Invitrogen^TM^, Waltham, MA, USA) and transferred to PVDF membranes. The membranes were then blocked with 5% skim milk diluted in 1X TBS-T containing 0.1% Tween-20 for 1 h at RT. To detect TET8 and PEN1, the membranes were incubated overnight at 4 °C with anti-TET8 (PHY1490A, PhytoAB, San Francisco, CA, USA) or anti-PEN1 (CSB-PA875527XA01DOA, CUSABIO, Houston, TX, USA) primary antibodies. Following three times washes with 1X TBS-T, the membranes were incubated with anti-rabbit horseradish peroxidase (HRP)-conjugated secondary antibody (PHY6000, PhytoAB, San Francisco, CA, USA) for 2 h at RT. Signal generation was achieved using the WestGlow™ FEMTO Chemiluminescent substrate (BIOMAX, Seoul, Republic of Korea). Images were captured using a ChemiDoc^TM^ MP Imaging System (BIO-RAD, Hercules, CA, USA).

### 2.5. Cell Culture

Human fibroblast cell lines (HFF and CCD-986Sk) were purchased from the American Type Culture Collection (ATCC, Manassas, VA, USA) and cultured in Dulbecco’s modified Eagle medium (DMEM; Welgene, Gyeongsan, Republic of Korea), supplemented with 10% fetal bovine serum (FBS; Gibco, Waltham, MA, USA) and 1% penicillin–streptomycin solution (P/S; Gibco, Waltham, MA, USA). Cells were maintained at 37 °C in a humidified incubator with 5% CO_2_.

### 2.6. WST-1 Assay for Cell Viability

For the cell viability assay, HFF and CCD-986Sk cells were each seeded at 3.0 × 10^3^ and 5.0 × 10^3^ cells/well in 96-well plates. The day after seeding, the cells were treated with EVs or miRNA mimics according to the experimental setup. Cells were incubated at 37 °C with 5% CO_2_ for 48 h. Cell viability was measured using the EZ-Cytox reagent (DoGENBIO, Seoul, Republic of Korea) following the manufacturer’s protocol. Absorbance was measured at 450 nm using a microplate reader (BioTek, Winooski, VT, USA).

### 2.7. In Vitro Wound Healing Assay

HFF and CCD-986Sk cells were each seeded at a density of 1.0 × 10^5^ and 3.0 × 10^5^ cells/well in 24-well plates. The day after seeding, the cells were scratched using an SPLScar™ Scratcher (SPL, Pocheon, Republic of Korea), and the cells were washed with DPBS to remove detached cells and debris. EVs or miRNA mimics were then treated with serum-free medium according to the experimental conditions. After 24 h of incubation at 37 °C with 5% CO_2_, the cells were fixed with 4% paraformaldehyde for 20 min at RT and stained with Coomassie-blue solution for 10 min at RT. Finally, the staining solution was removed, and the cells were washed with DPBS at least two times. Wound size was detected using a microscope (Nikon, Tokyo, Japan) and calculated using ImageJ software (version 1.54i).

### 2.8. Immunofluorescence (IF)

HFF cells were seeded at a density of 2.0 × 10^5^ cells/well in 6-well plates. The day after seeding, cells were irradiated with 1000 mJ/cm^2^ of UVA and treated with EVs or miRNA mimics for each experiment. After 48 h of incubation at 37 °C with 5% CO_2_, the cells were fixed with 4% paraformaldehyde for 20 min at RT and permeabilized with 0.1% Triton X-100 for 20 min at RT. Subsequently, the cells were blocked with 5% bovine serum albumin (BSA) diluted in PBS for 1 h at RT and incubated with COL1A1 antibody (ab316222, Abcam, Cambridge, UK) overnight at 4 °C. The following day, the cells were incubated with an Alexa Fluor 488-conjugated anti-rabbit secondary antibody (Abcam, Cambridge, UK) for 2 h at RT, followed by staining with 1 μg/mL DAPI for 15 min. Finally, the cells were washed with DPBS, and images were captured using a fluorescent microscope (Nikon, Tokyo, Japan). Green fluorescence for COL1A1 was normalized to blue fluorescence for DAPI using the ImageJ software program.

### 2.9. Quantitative Reverse-Transcription PCR (qRT-PCR)

Total RNAs were isolated using the XENOPURE^TM^ Small RNA Purification Kit (Xenohelix, Incheon, Republic of Korea) according to the manufacturer’s protocol, and RNA concentration was determined using a Nanodrop (Thermo Fisher Scientific, Waltham, MA, USA). Complementary DNA (cDNA) was synthesized using a cDNA synthesis kit (GenDEPOT, Baker, CA, USA) according to the manufacturer’s protocol. The expression of target genes was detected using SYBR^®^ Green Master Mix (Bio-Rad, Hercules, CA, USA) via a qRT-PCR machine (Bio-Rad, Hercules, CA, USA). All genes were normalized to GAPDH and calculated using the 2^(−ΔΔCq)^ method. For miRNA expression analysis, cDNA synthesis and qRT-PCR were performed using the Mir-X First-Strand Synthesis Kit and Mir-X qRT-PCR TB Green Kit (Takara, Osaka, Japan), according to the manufacturer’s protocol. The expression of miRNA was normalized to U6 and calculated using the 2^(−ΔΔCq)^ method. The primer sequences used in this study are listed in Table S1.

### 2.10. Small RNA-Sequencing

Small RNA sequencing was performed by Xenohelix (Republic of Korea). Small RNAs were extracted from Cm-callus EVs using XENO-EVARI^TM^ Kit (Xenohelix, Incheon, Republic of Korea), and a small RNA library was generated using XENO-LIBERA library kit (Xenohelix, Incheon, Republic of Korea) following the manufacturer’s protocol. The resulting library was sequenced using the NextSeq 500 system (Illumina, San Diego, CA, USA). Quality control of raw data for sequencing was conducted using FastQC v0.11.9 [21]. The adapter sequences and low quality of 3′ end sequences (quality score < 20) were removed, and remaining reads were trimmed based on length (18~30 nucleotides) using Cutadapt v4.4 [22]. These trimmed data were further processed to isolate reads of non-coding RNA (ncRNA) aligned with Viridiplantae ncRNA sequences (data from Rfam) using bowtie v1.3.1 [23]. Among these extracted data, reads with 20~22 nucleotides were predicted as miRNAs using BrumiR v3.0 [24], and the expression of miRNAs was quantified using bowtie v1.3.1 [23]. Finally, the family of predicted miRNAs was predicted using the miRNA database from miRbase [25] and the Blast v2.2.29 software program [26].

### 2.11. Transfection of miRNA Mimics

To overexpress miRNA, HFF and CCD-986Sk cells were transfected with mimics of miR167 or a negative control (N.C.). Cells were cultured in 6-well plates and transfected with 10 nM or 20 nM of mimics using opti-MEM medium and RNAiMAX Transfection Reagent (Invitrogen, Carlsbad, CA, USA) according to the manufacturer’s protocol. The sequences of the mimics used in this study are presented in Table S2. The N.C. mimic (Cat # SMC-2002) was purchased from Bioneer (Daejeon, Republic of Korea).

### 2.12. Statistical Analysis

All experiments were performed at least 3 times, and the data are presented as the mean ± standard deviation (SD). Statistical significance was assessed using ordinary one-way ANOVA, followed by Student’s *t*-test, with a threshold of *p* ≤ 0.05 considered statistically significant. All reported data are presented as mean ± standard error (SE), unless stated otherwise.

## 3. Results

### 3.1. Induction of Callus from C. maritimum

**Figure 1:**
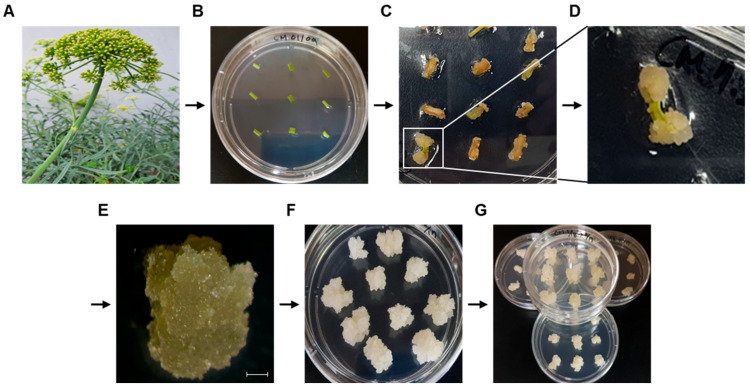
Cm-callus induction from *Crithmum maritimum.* (**A**) Raw materials of the rock samphire (*Crithmum maritimum*) plant. (**B**) Leaf and stem explants on callus induction medium (CIM). (**C**,**D**) Induced callus after 4 weeks and enlarged image. (**E**) Selected callus after three subcultures on CIM (Scale bar; 1 mm). (**F**,**G**) Homogeneous callus clusters.

**Figure 2:**
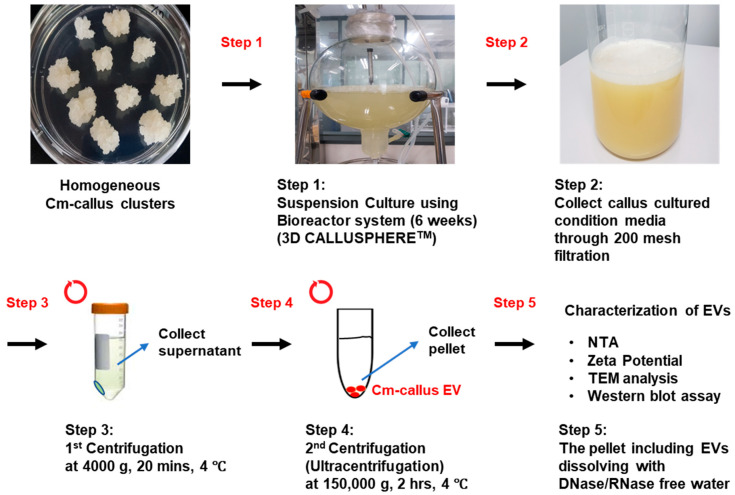
Isolation and identification of Cm-callus-derived extracellular vesicles (Cm-callus EVs).

Callus induction from *C. maritimum* (Cm-callus) was achieved in approximately 30–70% of explants, depending on the type and concentration of auxin hormone used. Explants showed swelling and cell proliferation, especially at the edge of the cuttings (Figure 1A,B), which led to callus formation within 4 weeks (Figure 1C,D). To obtain homogeneous calluses, the calluses were subcultured on callus induction medium (CIM) three times at 4-week intervals (Figure 1E). We obtained a homogeneous bright white callus cell line with a friable texture suitable for propagation (Figure 1F,G). Callus clusters successfully cultivated on medium containing 0.3 mg/L 2,4-D were used to establish a suspension culture (Figure 2). This process was initiated in a 1 L Erlenmeyer flask containing 400 mL of the medium and shaken at 120–150 rpm. The culture was subsequently scaled up in a bioreactor by a 10-fold increase in volume, with regulated airflow, and maintained under these conditions for an additional 6 weeks.

### 3.2. Isolation and Characterization of Extracellular Vesicles (EVs) from Callus of C. maritimum

**Figure 3:**
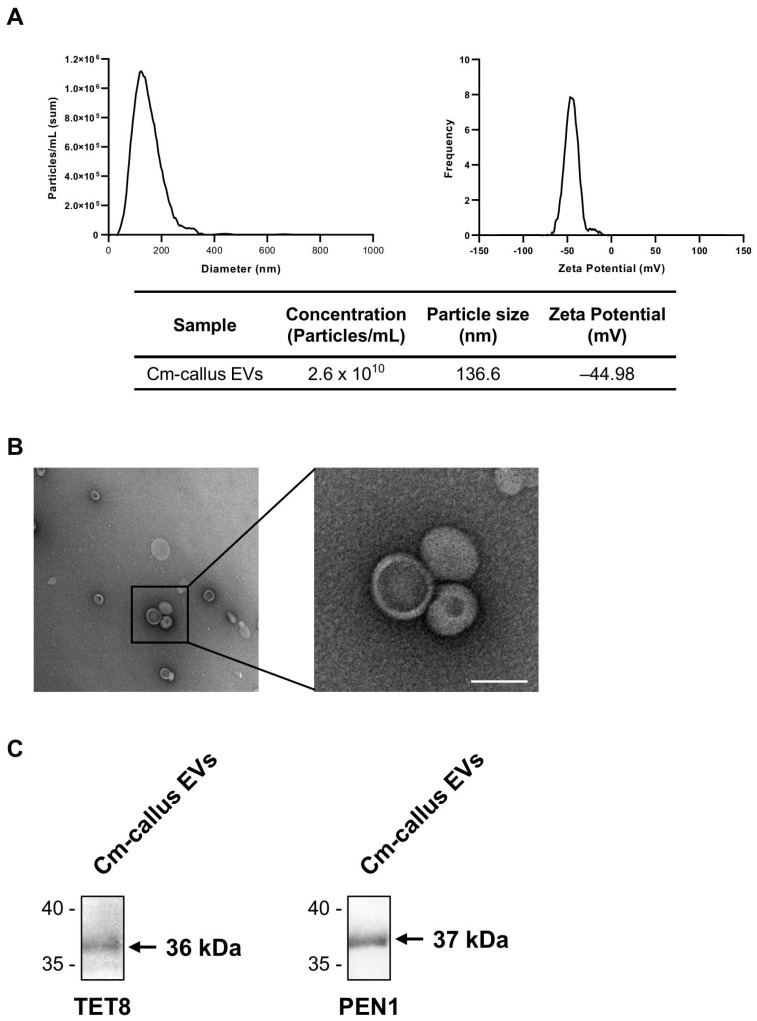
Characterization of Cm-callus EVs. (**A**) Nanoparticle tracking analysis (left) and Zeta Potential (right) of Cm-callus EVs, resultingin 2.6 × 1010 particles/mL of concentration, 136.6 nm of mean size, and −44.98 mV of zeta potential. (**B**) Transmission electron microscopy images of Cm-callus EVs. (Scale Bar; 100 nm) (**C**) Western Blot assay of TET8, PEN1 expressions in Cm-callus EVs, detected at 36 and 37 kDa, respectively.

EVs from Cm-callus (Cm-callus EVs) were isolated and purified according to the workflow shown in Figure 2. The characteristics of Cm-callus EVs were confirmed by analyzing their biophysical properties. The size and concentration of Cm-callus EVs, measured using nanoparticle tracking analysis (NTA), were 136.6 nm and 2.6 × 10^10^ particles/mL (Figure 3A). The zeta potential of Cm-callus EVs, measured using ZetaView, was −44.98 mV (Figure 3A). The morphology of Cm-callus EVs, detected by transmission electron microscopy (TEM), was round, with a diameter of up to 200 nm (Figure 3B). The expression of TET8 and PEN1, a well-known biomarker of plant-derived extracellular vesicles, was analyzed using western blot (Figure 3C). These results confirmed that the substances isolated from Cm-callus were indeed EVs.

### 3.3. Effect of Cm-callus EVs on Wound Healing and Skin Regeneration in Human Fibroblasts

**Figure 4:**
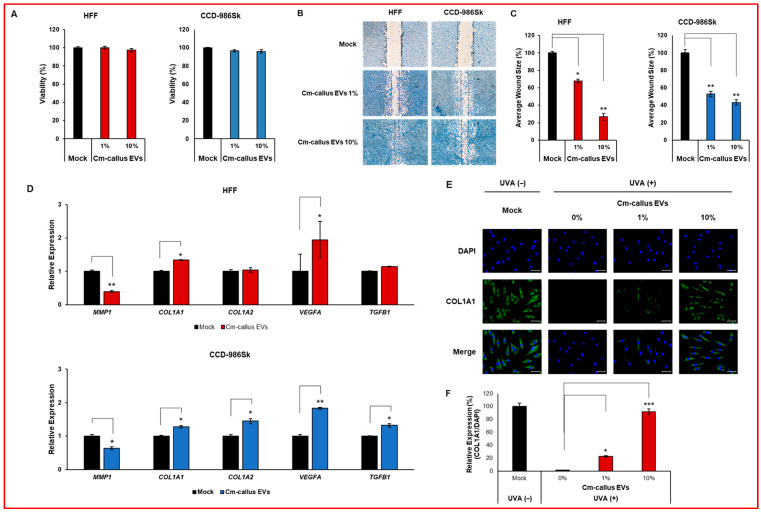
In vitro functional characterization of Cm-callus EVs. (**A**) No significant changes in the viability of human fibroblasts after treatment with Cm-callus EVs. (**B**,**C**) Decreased average wound size in wounded human fibroblasts dose-dependently after treatment with Cm-callus EVs (**B**) and evaluation of average wound size in [%] (**C**). (**D**) Changes in mRNA expression of *MMP1*, *COL1A1*, *COL1A2*, *VEGFA*, and *TGFB1* genes in human fibroblasts following treatment with Cm-callus EVs. (**E**,**F**) Changes and recovery in protein expression of COL1A1 in HFF cells after treatment with Cm-callus EVs following UV irradiation (**E**) and evaluation of relative expression of COL1A1 normalized with DAPI (**F**). (green: COL1A1 labeled by Alexa Fluor 488; blue: nucleus labeled by DAPI, Scale bar: 50 μm), * *p* < 0.05, ** *p* < 0.01, *** *p* < 0.001.

To assess the biological efficacy of Cm-callus EVs, a cell viability assay was first conducted to determine whether exposure to varying concentrations of Cm-callus EVs affected the viability of human fibroblast cell lines. No significant differences in cell viability were observed across the different concentrations of the tested EVs (Figure 4A). To determine whether Cm-callus EVs affect wound healing, wounded human fibroblasts were treated with 1% or 10% of Cm-callus EVs. Treatment with Cm-callus EVs led to a dose-dependent decrease in the average wound size compared to the control (Mock) in both cell lines (Figure 4B,C). Skin regeneration after injury is a vital and complex process regulated by numerous factors, including the expression of key genes such as *MMP1*, *COL1A1*, *COL1A2*, *VEGFA*, and *TGFB1*. Changes in mRNA expression of these genes in human fibroblasts were analyzed using qRT-PCR. In both cell lines, treatment with Cm-callus EVs resulted in a significant reduction in *MMP1* expression. However, the expression of *COL1A1*, *COL1A2*, *VEGFA*, and *TGFB1* was notably upregulated by treatment with Cm-callus EVs, except for the expression of *COL1A2* and *TGFB1* in HFF cells, compared to that in the untreated (mock) control group (Figure 4D). To assess the effect of Cm-callus EVs on COL1A1 protein expression, immunofluorescence (IF) was performed on HFF cells. The expression of COL1A1 was reduced by UV irradiation and recovered in a dose-dependent manner upon treatment with Cm-callus EVs, with recoveries of 23% and 92% at 1% and 10% concentrations, respectively (Figure 4E,F). These results suggest that Cm-callus EVs promote skin regeneration without inducing cytotoxicity.

### 3.4. Enrichment of tae-miR167c-5p in Cm-callus EVs

**Figure 5:**
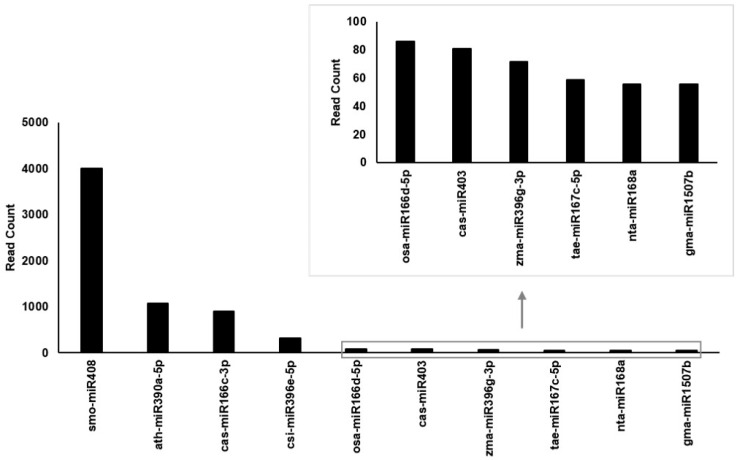
Candidates of miRNAs present in Cm-callus EVs. Top 10 candidates of miRNAs with the highest number of read counts in Cm-callus EVs, including miR167 (tae-miR167c-5p), those with a read count of less than 100 are enlarged.

EVs contain various biomolecules, including DNA, RNA, and proteins. miR167 is associated with salinity tolerance in halophytic plants. Therefore, this study focused on miRNAs to investigate whether miR167 is present in Cm-callus EVs. Small RNA sequencing was performed using small RNAs extracted from Cm-callus EVs. Cm-callus EVs had 10,194 miRNA reads out of 4,787,527 total reads (Table 1). To increase data reliability, reads were filtered based on the e-value (<1 × 10^−5^) and bitscore (>35), resulting in the identification of the top 10 miRNA candidates (Figure 5 and Table S3). Among these, tae-miR167c-5p (miR167) ranked 8th, suggesting that miR167 may be a key factor contributing to the wound healing efficacy of Cm-callus EVs.

### 3.5. Effect of miR167 from Cm-callus EVs on Wound Healing and Skin Regeneration in Human Fibroblasts

**Figure 6:**
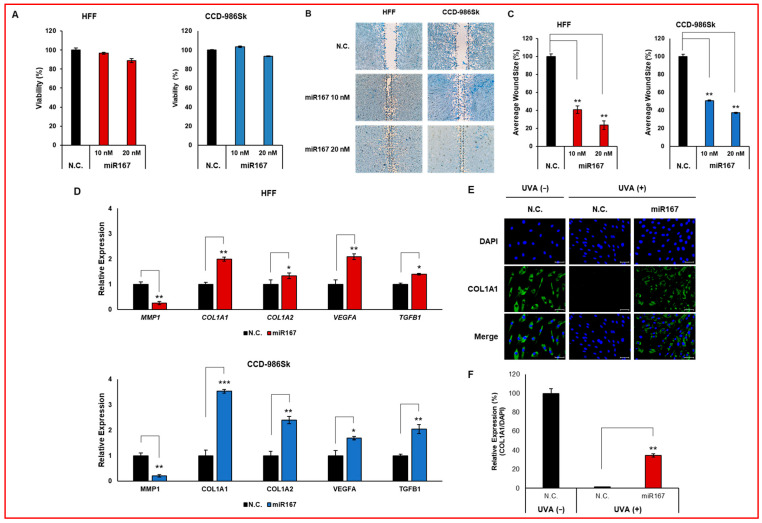
In vitro functional characterization of miR167. (**A**) No significant changes in the viability of human fibroblasts after transfection with the miR167 mimic. (**B**,**C**) Decreased average wound size in wounded human fibroblasts in a dose-dependent manner following transfection with the miR167 mimic (**B**) and evaluation of average wound size in [%] (**C**). (**D**) Changes in mRNA expression of *MMP1*, *COL1A1*, *COL1A2*, *VEGFA*, and *TGFB1* genes in human fibroblasts after transfection with the miR167 mimic. (**E**,**F**) Changes and recovery in the protein expression of COL1A1 in HFF cells by transfection with the miR167 mimic after UV irradiation (**E**) and evaluation of the relative expression of COL1A1 normalized with DAPI (**F**). (green: COL1A1 labeled by Alexa Fluor 488; blue: nucleus labeled by DAPI, Scale bar: 50 μm), * *p* < 0.05, ** *p* < 0.01, *** *p* < 0.001.

To determine whether miR167 derived from Cm-callus EVs mimics the wound-healing efficacy of Cm-callus EVs, the viability of human fibroblasts was assessed after transfection with an miR167 mimic. Cell viability remained unaffected across different doses of the miR167 mimic (Figure 6A), demonstrating high transfection efficiency, with miR167 expression increasing by approximately 12,000- and 6000-fold in both cell lines, respectively (Figure S1). Notably, transfection with miR167 mimic resulted in a dose-dependent reduction in wound size in human fibroblasts compared to control treated with N.C. mimic (Figure 6B,C). Additionally, the changes in the mRNA expression of wound healing-related genes, such as *COL1A1*, *COL1A2*, *MMP1*, *VEGFA*, and *TGFB1*, induced by the miR167 mimic were consistent with those observed following Cm-callus EVs treatment (Figure 6D), except for *COL1A2* and *TGFB1* expression in HFF cells. Specifically, in HFF cells, the expression of *MMP1* decreased by approximately 74%, whereas *COL1A1*, *COL1A2*, *VEGFA*, and *TGFB1* increased by 2.0-, 1.3-, 2.1-, and 1.4-fold, respectively. Similarly, in CCD-986Sk cells, the expression of *MMP1* decreased by approximately 79%, whereas *COL1A1*, *COL1A2*, *VEGFA*, and *TGFB1* were increased by 3.5-, 2.4-, 1.7-, and 2.0-fold, respectively. Immunofluorescence (IF) was performed to evaluate the effect of miR167 on COL1A1 protein expression in HFF cells. UV irradiation significantly reduced COL1A1 expression, which was restored by transfection with an miR167 mimic. This restoration resulted in an approximate recovery of 35% compared to transfection with an N.C. mimic (Figure 6E,F). These results indicate that miR167 promotes skin regeneration without inducing cell cytotoxicity, exhibiting effects similar to those of Cm-callus EVs.

### 3.6. PPP3R2 Could be a Target of miR167

miRNAs regulate target mRNAs by inducing mRNA cleavage or inhibiting their translation. This study revealed that miR167, derived from Cm-callus EVs, influences skin regeneration. However, the specific target of this miRNA has yet to be identified. To address this issue, potential targets were predicted using psRNATarget V1 and V2 [27,28]. From V2, 96 candidate targets were identified (Table 2 and Table S4), whereas V1 identified only five candidates (Table S5). In these results, the term “expectation” refers to the penalty for mismatches between mature small RNAs and the target sequence. “UPE” means “unpaired energy” as the target accessibility, which is represented by the energy required to open the secondary structure around the target site on the target mRNA. The higher the expectation, the lower the similarity (and possibility) between the small RNA and the target candidate, and the lower the UPE, the greater the possibility that the small RNA can contact (and cleave) the target mRNA. By integrating the results from both prediction tools, the *PPP3R2* gene emerged as a potential target of miR167. *PPP3R2* is associated with the mitogen-activated protein kinase (MAPK) and nuclear factor of activated T-cells (NFAT) pathways, both of which are implicated in skin regeneration. However, these findings are based on computational predictions and have not yet been experimentally validated. To elucidate the precise mechanism of the ‘miR167-target gene-skin regeneration’ axis, further experimental studies are required.

## 4. Discussion

PDEVs are nanosized (30–150 nm) membrane vesicles that carry biomolecules that influence plant development and protect plants against pathogens [11,29]. In addition, PDEVs have been shown to exchange information with mammals across kingdoms [29]. Recently, PDEVs have gained attention as novel, biologically active materials for skin care [6]. The zeta potential and size of EVs are critical factors for evaluating their stability and effectiveness as drug delivery systems [30]. Cm-callus EVs exhibit a zeta potential of −44.98 mV and an average size of 136.6 nm (Figure 3A), characteristics that are particularly advantageous for their application as drug delivery systems. The zeta potential, which indicates the surface charge, reflects a strong negative charge in Cm-callus EVs, which promotes electrostatic repulsion between vesicles, ensuring excellent colloidal stability and preventing aggregation [31]. Additionally, a negative charge may improve cellular uptake by facilitating interactions with positively charged components of the cell membrane, which is a key factor in effective drug delivery [32]. The average size of Cm-callus EVs (136.6 nm) fell within the optimal range (100–200 nm) for drug delivery systems. Nanoparticles of this size are efficiently internalized by cells through endocytic pathways such as clathrin-mediated endocytosis, enabling targeted therapeutic delivery [33,34]. Their size also contributes to their favorable biodistribution and prolonged retention in the bloodstream [35]. Moreover, nanoparticles under 150 nm can exploit the enhanced permeability and retention (EPR) effect, allowing passive accumulation in tissues with leaky vasculature, such as inflamed or tumor regions, further enhancing their targeting capabilities [36]. Taken together, the combination of a highly negative zeta potential and optimal size of Cm-callus EVs positions them as a promising platform for drug delivery. These properties not only ensure high stability and efficient cellular uptake but also provide the potential to encapsulate and protect bioactive molecules, target specific tissues, and minimize systemic toxicity. These characteristics underline the significant potential of Cm-callus EVs for advancing therapeutic applications and warrant further exploration of their functionalization and clinical use, making them highly suitable for drug delivery.

In this study, we demonstrated the role of Cm-callus EVs in promoting skin regeneration. The regenerative capability of Cm-callus EVs was validated using a wound-healing assay. Treatment of fibroblasts with Cm-callus EVs reduced the wound size by up to 70% (Figure 4B,C). As wound healing and skin regeneration progressed, the expression of *MMP1* and *MMP3* was downregulated, along with an upregulation of *COL1A1*, *COL1A2*, *VEGFA*, and *TGFB1* expression [37,38]. These trends were generally consistent with the observed downregulation of *MMP1* mRNA and upregulation of *COL1A1*, *COL1A2*, *VEGFA*, and *TGFB1* mRNA in fibroblasts treated with Cm-callus EVs (Figure 4D), although some exceptions were noted in HFF cells. Specifically, the expression levels of *COL1A2* and *TGFB1* did not show significant changes in HFF cells after treatment. This discrepancy may result from cell type-specific regulatory mechanisms or the presence of additional bioactive components within EVs. However, the changes observed in the expression of the other genes may provide valuable insights into the mechanism of skin regeneration. Notably, the UV-induced reduction in COL1A1 protein expression was reversed following treatment with Cm-callus EVs (Figure 4E,F). These findings strongly suggest that Cm-callus EVs promote skin regeneration.

To further elucidate the specific components of EVs responsible for their regenerative effects, we focused on miRNAs, which are key biomolecules in EVs. miR167, a well-studied miRNA known to confer salt tolerance to halophytes, is of particular interest [19,20]. Small RNA sequencing (sRNA-seq) revealed that Cm-callus EVs contained more than 100 miRNAs, including miR167 (Figure 5). Functional analyses confirmed that miR167 possessed skin-regenerative properties comparable to or even exceeding those of Cm-callus EVs. Transfection of fibroblasts with the miR167 mimic reduced the wound size by up to 80% (Figure 6B,C). Additionally, the gene expression changes associated with skin regeneration following miR167 transfection were consistent with those observed after Cm-callus EV treatment (Figure 6D–F), except for certain genes in HFF cells. These results suggest that miR167 plays a pivotal role in mediating the skin regenerative effects of Cm-callus EVs.

miRNAs regulate target mRNAs through mechanisms such as mRNA degradation and translation inhibition. In this study, we investigated the potential targets of miR167, an miRNA enriched in Cm-callus EVs, using the small RNA target analysis server psRNATarget [27]. Our results identified approximately 100 candidate target genes, with *PPP3R2* being the top candidate. Notably, *PPP3R2* was highly ranked in both versions of the prediction tool, indicating a strong potential for miR167 to regulate its expression. PPP3R2 is associated with both the MAPK and NFAT signaling pathways, which are critical for various cellular processes, including skin regeneration and repair. The MAPK pathway plays an essential role in regulating cell proliferation, differentiation, and survival, which are crucial for wound healing and tissue regeneration [39,40]. The NFAT pathway, on the other hand, is involved in immune response regulation and has been shown to influence collagen production, a key factor in skin elasticity and structure [41,42]. Both pathways contribute to maintaining skin integrity by modulating fibroblast function, collagen synthesis, and inflammatory responses, which are essential for combating the signs of aging. The prediction that miR167 targets *PPP3R2*, a gene involved in relevant signaling pathways, presents a compelling possibility for its anti-aging mechanisms. By modulating *PPP3R2* expression, miR167 may influence the MAPK and NFAT pathways, thereby potentially enhancing skin regeneration and decelerating the aging process. This aligns with the regenerative effects observed with Cm-callus EVs treatment in the fibroblast wound healing assay, where key genes involved in collagen synthesis (such as *COL1A1* and *COL1A2*) were upregulated, and markers of tissue degradation, such as *MMP1*, were downregulated. Thus, the potential targeting of *PPP3R2* by miR167 may represent a mechanism through which Cm-callus EVs promote skin regeneration and exhibit anti-aging effects. Although our findings provide strong computational evidence linking miR167 to *PPP3R2* and the regulation of important signaling pathways in skin regeneration, further experimental studies are required to validate these predictions. Specifically, functional confirmation of *PPP3R2* as a target of miR167 and its involvement in skin regeneration processes are key to fully understanding the therapeutic potential of miRNA-based treatments in anti-aging skincare.

## 5. Conclusions

In conclusion, this study provides evidence that Cm-callus EVs, particularly their content of miR167, promote skin regeneration by modulating the key genes involved in tissue repair and collagen synthesis. Although the exact molecular mechanisms remain to be elucidated, our findings support the potential of plant-derived EVs as a novel strategy for enhancing skin regeneration, with miR167 playing a pivotal role. Further studies are needed to validate the targets of miR167 and explore the therapeutic applications of Cm-callus EVs in skin regeneration.

## Figures and Tables

**Table 1:** Results of small RNA sequencing of Cm-callus EVs.

Sample	# Total Reads	# Mapped Reads			
		miRNA *	rRNA †	tRNA †	ncRNA † (Other)
Cm-callus EVs	4,787,527	10,194	647,678	4900	3,284,785

# number of Reads. * mapped reads of miRNA: Summation of read counts for predicted miRNAs. † Reference data: Rfam database.

**Table 2:** Predicted target genes of miR167 (Top 10).

Putative Human Target Genes of tae-miR167c-5p (from. psRNATarget V2)					
Rank	Target Accession	Expectation	UPE	mRNA Target Aligned Fragment (5′-3′)	Inhibition
1	NM_147180|PPP3R2	3	−1	UUAUGUCAUGUUGGUAGCUUUA	Cleavage
2	NM_001122853|MYOZ3	3	−1	GAUGAUGAUGAUGGCAGCUUUA	Cleavage
3	NM_006830|UQCR11	3.5	−1	GUUGAUCAUGCUGGUGGCUUGG	Cleavage
4	NM_012479|YWHAG	3.5	−1	AUGGAUCGUGUUGGUAUUUUCA	Cleavage
5	NM_015026|MON2	3.5	−1	UAUGAUCAUGCAGUUAGCUUCA	Translation
6	NM_080391|PTP4A2	4	−1	UCAGAGAAUGCUGGUAGCUUAA	Cleavage
7	NM_022340|ZFYVE20	4	−1	CAGGAUCGUGCUGGUAGCACCA	Cleavage
8	NM_003672|CDC14A	4	−1	GGGAAUCAUGUUGACAGUUUUA	Cleavage
9	NM_006004|UQCRH	4	−1	UUGGCUUAGGCUGGUAGCUUCU	Cleavage
10	NM_001089591|UQCRHL	4	−1	UUGGCUUAGGCUGGUAGCUUCU	Cleavage

## Data Availability

The data that support the findings of this study are available from the corresponding author upon reasonable request.

## Supplementary Materials

The following supporting information can be downloaded at: https://www.mdpi.com/article/10.3390/biom15081157/s1. Figure S1: Transfection efficiency of miR167; Table S1: Sequences of primers for qRT-PCR; Table S2: Sequence of mimic for transfection; Table S3: Result of sRNA-seq for Cm-callus EVs; Tables S4 and S5: Putative human target genes of tae-miR167c-5p.

## References

[1] Flowers T.J., Colmer T.D. (2008). Salinity tolerance in halophytes*. New Phytol..

[2] Mzoughi Z., Majdoub H. (2021). Pectic polysaccharides from edible halophytes: Insight on extraction processes, structural characterizations and immunomodulatory potentials. Int. J. Biol. Macromol..

[3] Zengin G., Aumeeruddy-Elalfi Z., Mollica A., Yilmaz M.A., Mahomoodally M.F. (2018). In vitro and in silico perspectives on biological and phytochemical profile of three halophyte species—A source of innovative phytopharmaceuticals from nature. Phytomedicine.

[4] Radman S., Mastelić L., Ljubenkov I., Lazarevski S., Politeo O., Podrug R., Prga I., Čorić I., Popović M., Bratinčević M.V. (2024). Sea Fenel (*Crithmum maritimum* L.) Flowers as an Emerging Source of Bioactive Compounds Pol. J. Food Nutr. Sci..

[5] Generalić Mekinić I., Blažević I., Mudnić I., Burčul F., Grga M., Skroza D., Jerčić I., Ljubenkov I., Boban M., Miloš M. (2016). Sea fennel (*Crithmum maritimum* L.): Phytochemical profile, antioxidative, cholinesterase inhibitory and vasodilatory activity. J. Food Sci. Technol..

[6] Subha D., Harshnii K., Madhikiruba K.G., Nandhini M., Tamilselvi K.S. (2023). Plant derived exosome-like Nanovesicles: An updated overview. Plant Nano Biol..

[7] O’Brien K., Breyne K., Ughetto S., Laurent L.C., Breakefield X.O. (2020). RNA delivery by extracellular vesicles in mammalian cells and its applications. Nat. Rev. Mol. Cell Biol..

[8] Bongiovanni1 L., Andriessen A., Wauben M.H.M., Nolte-‘t Hoen E.N.M., Bruin A. (2021). Extracellular Vesicles: Novel Opportunities to Understand and Detect Neoplastic Diseases. Vet. Pathol..

[9] Wang Y., Wang J., Ma J., Zhou Y., Lu R. (2022). Focusing on Future Applications and Current Challenges of Plant Derived Extracellular Vesicles. Pharmaceuticals.

[10] Ju S., Mu J., Dokland T., Zhuang X., Wang Q., Jiang H., Xiang X., Deng Z.-B., Wang B., Zhang L. (2013). Grape Exosome-like Nanoparticles Induce Intestinal Stem Cells and Protect Mice from DSS-Induced Colitis. Mol. Ther..

[11] Alzahrani F.A., Khan M.I., Kameli N., Alsahafi E., Riza Y.M. (2023). Plant-Derived Extracellular Vesicles and Their Exciting Potential as the Future of Next-Generation Drug Delivery. Biomolecules.

[12] Gu S., Jin L., Zhang F., Sarnow P., Kay M.A. (2009). The biological basis for microRNA target restriction to the 3′ untranslated region in mammalian mRNAs. Nat. Struct Mol. Biol..

[13] Dong Q., Hu B., Zhang C. (2022). microRNA and Their Roles in Plant Development. Front. Plant Sci..

[14] Saiyed A.N., Vasavada A.R., Kaid Johar S.R. (2022). Recent trends in miRNA therapeutics and the application of plant miRNA for prevention and treatment of human diseases. Futur. J. Pharm Sci..

[15] Wu M.F., Tian Q., Reed J.W. (2006). *Arabidopsis* microRNA167 controls patterns of *ARF6* and *ARF8* expression, and regulates both female and male reproduction. Development.

[16] Liu X., Huang S., Xie H. (2021). Advances in the regulation of plant development and stress response by miR167. Front. Biosci..

[17] Díez-Sainz E., Milagro F.I., Aranaz P., Riezu-Boj J.I., Lorente-Cebrián S. (2024). MicroRNAs from edible plants reach the human gastrointestinal tract and may act as potential regulators of gene expression. J. Physiol. Biochem..

[18] Philip A., Ferro V.A., Tate R.J. (2015). Determination of the potential bioavailability of plant microRNAs using a simulated human digestion process. Mol. Nutr. Food Res..

[19] Islam W., Waheed A., Naveed H., Zeng F. (2022). MicroRNAs Mediated Plant Responses to Salt Stress. Cells.

[20] Gharat S.A., Shaw B.P. (2015). Novel and conserved miRNAs in the halophyte *Suaeda maritima* identified by deep sequencing and computational predictions using the ESTs of two mangrove plants. BMC Plant Biol..

[21] de Sena Brandine G., Smith A.D. (2019). Falco: High-speed FastQC emulation for quality control of sequencing data. F1000Research.

[22] Martin M. (2011). Cutadapt removes adapter sequences from high-throughput sequencing reads. EMBnet. J..

[23] Langmead B., Trapnell C., Pop M., Salzberg S.L. (2009). Ultrafast and memory-efficient alignment of short DNA sequences to the human genome. Genome Biol..

[24] Moraga C., Sanchez E., Ferrarini M.G., Gutierrez R.A., Vidal E.A., Sagot M.F. (2022). BrumiR: A toolkit for de novo discovery of microRNAs from sRNA-seq data. GigaScience.

[25] Griffiths-Jones S., Grocock R.J., van Dongen S., Bateman A., Enright A.J. (2006). miRBase: microRNA sequences, targets and gene nomenclature. Nucleic Acids Res..

[26] Camacho C., Coulouris G., Avagyan V., Ma N., Papadopoulos J., Bealer K., Madden T.L. (2009). BLAST+: Architecture and applications. BMC Bioinform..

[27] Dai X., Zhuang Z., Zhao P.X. (2018). psRNATarget: A plant small RNA target analysis server (2017 release). Nucleic Acids Res..

[28] Díez-Sainz E., Aranaz P., Amri E.-Z., Riezu-Boj J.I., Lorente-Cebrián S., Milagro F.I. (2024). Plant miR8126-3p and miR8126-5p Decrease Lipid Accumulation Through Modulation of Metabolic Genes in a Human Hepatocyte Model That Mimics Steatosis. Int. J. Mol. Sci..

[29] Cho J.H., Hong Y.D., Kim D.H., Park S.J., Kim J.S., Kim H.-M., Yoon E.J., Cho J.-S. (2022). Confrmation of plant-derived exosomes as bioactive substances for skin application through comparative analysis of keratinocyte transcriptome. Appl. Biol. Chem..

[30] Midekessa G., Godakumara K., Ord J., Viil J., Lättekivi F., Dissanayake K., Kopanchuk S., Rinken A., Andronowska A., Bhattacharjee S. (2020). Zeta Potential of Extracellular Vesicles: Toward Understanding the Attributes that Determine Colloidal Stability. ACS Omega.

[31] Pochapski D.J., dos Santos C.C., Leite G.W., Pulcinelli S.H., Santilli C.V. (2021). Zeta Potential and Colloidal Stability Predictions for Inorganic Nanoparticle Dispersions: Effects of Experimental Conditions and Electrokinetic Models on the Interpretation of Results. Langmuir.

[32] Öztürk K., Kaplan M., Çalış S. (2024). Effects of nanoparticle size, shape, and zeta potential on drug delivery. Int. J. Pharm..

[33] Tang X.R., Lei S.-Y., Zhang Q., Liu Y.-Y., Wu H., Cao A., Wang H. (2025). How big nanoparticles carry small ones into cells: Actions captured by transmission electron microscopy. Colloids Surf. B Biointerfaces.

[34] Rennick J.J., Johnston A.P.R., Parton R.G. (2021). Key principles and methods for studying the endocytosis of biological and nanoparticle therapeutics. Nat. Nanotechnol..

[35] Hoshyar N., Gray S., Han H., Bao G. (2016). The effect of nanoparticle size on in vivo pharmacokinetics and cellular interaction. Nanomedicine.

[36] Prabhakar U., Maeda H., Jain R.K., Sevick-Muraca E.M., Zamboni W., Farokhzad O.C., Barry S.T., Gabizon A., Grodzinski P., Blakey D.C. (2013). Challenges and Key Considerations of the Enhanced Permeability and Retention Effect for Nanomedicine Drug Delivery in Oncology. Cancer Res..

[37] Kunhorm P., Chaicharoenaudomrung N., Noisa P. (2023). Cordycepin-induced Keratinocyte Secretome Promotes Skin Cell Regeneration. In Vivo.

[38] Lee Y.I., Lee S.G., Ham S.Y., Jung I.H., Suk J.M., Lee J.H. (2024). Exploring the Safety and Efficacy of Organic Light-Emitting Diode in Skin Rejuvenation and Wound Healing. Yonsei Med. J..

[39] Leyane T.S., Jere S.W., Houreld N.N. (2021). Cellular Signalling and Photobiomodulation in Chronic Wound Repair. Int. J. Mol. Sci..

[40] Cargnello M., Roux P.P. (2011). Activation and Function of the MAPKs and Their Substrates, the MAPK-Activated Protein Kinases. Microbiol. Mol. Biol. Rev..

[41] Lin Y., Song Y., Zhang Y., Shi M., Hou A., Han S. (2023). NFAT signaling dysregulation in cancer: Emerging roles in cancer stem cells. Biomed. Pharmacother..

[42] Manabe T., Park H., Minami T. (2021). Calcineurin-nuclear factor for activated T cells (NFAT) signaling in pathophysiology of wound healing. Inflamm. Regen..

