# Supplementary material for: The Role of miRNA167 in Skin Improvement: Insight from Extracellular Vesicles Derived from Rock Samphire (Crithmum maritimum)
Source: Biomolecules. 2025 Aug 12;15(8):1157. doi: 10.3390/biom15081157 (PMC12384170; doi:10.3390/biom15081157)
Supplement: Supplementary file 1 [file biomolecules-15-01157-s001.zip › biomolecules-3786859-supplementary.pdf]

Supplementary Data

The role of miRNA167 in skin improvement : Insight from extracellular vesicles derived from Rock Samphire (*Crithmum maritimum*)

Soll Jin[1,2,†], ChangHoe Ku[3,†], Hye Jin Kim[1], Jae-Goo Kim[1], Sang Hoon Kim[2], Heyjin Han[4], Hee Cheol Kang[1], Jae Sung Hwang[3,*], and Mi Jung Kim[1,*]

[1]Human & Microbiome Communicating Laboratory, GFC Co., Ltd., Hwaseong 18471, Republic of Korea; [2]Department of Biology, Kyung Hee University, Seoul 02447 Republic of Korea; [3]Department of Genetics & Biotechnology, Graduate School of Biotechnology, College of Life Sciences, Kyung Hee University, Yongin 17104, Republic of Korea; [4]GRIDA, Anseong 17577, Republic of Korea;

[†] Both authors contributed equally to this work

*Correspondence: jshwang@khu.ac.kr (J.S.Hwang), mj2.kim@gfcos.co.kr (M.J. Kim)

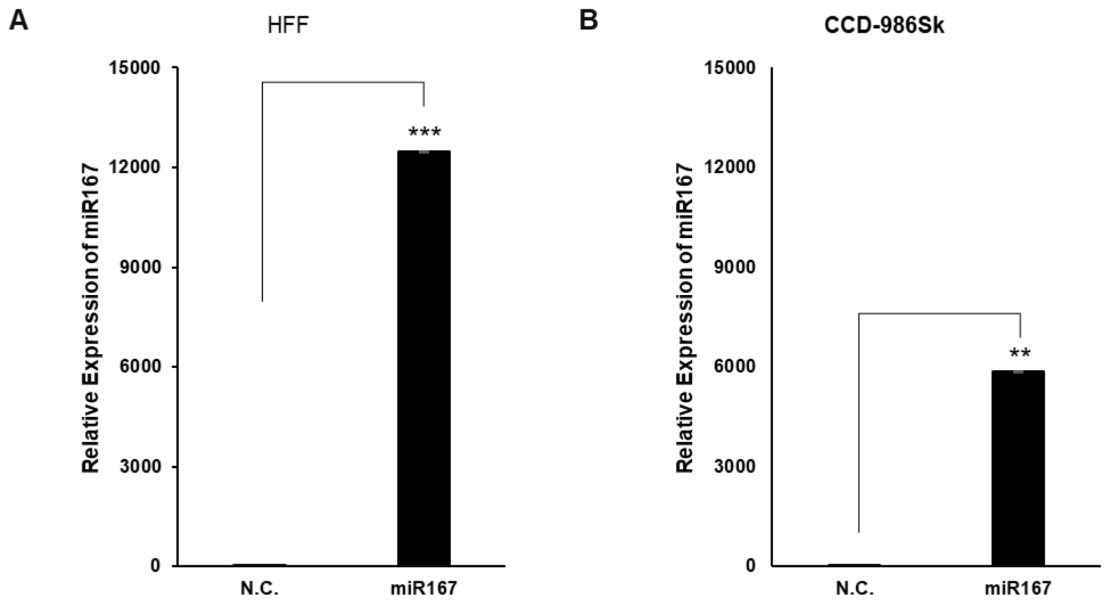

Figure S1. Transfection efficiency of miR167

qRT-PCR analysis for transfection efficiency of miR167 mimic into HFF cells (A) and CCD-986Sk cells (B), $^{**}p<0.01$, $^{***}p<0.001$.

Table S1. Sequences of primers for qRT-PCR

Primer	strand	Sequence (5' – 3')
**hMMP1**	Forward	CCCAGCGACTCTAGAAACAC
	Reverse	GCCTCCCATCATTCTTCAGG
**hCOL1A1**	Forward	GATTCCCTGGACCTAAAGGTGC
	Reverse	AGCCTCTCCATCTTTGCCAGCA
**hCOL1A2**	Forward	GGTGAAGTGGGTCTTCCAGG
	Reverse	TAAGGCCGTTTGCTCCAGG
**hVEGF**	Forward	ACGGACAGACAGACAGACAC
	Reverse	GAAGCGAGAACAGCCCAGAA
**hTGFβ**	Forward	TACCTGAACCCGTGTTGCTCTC
	Reverse	GTTGCTGAGGTATCGCCAGGAA
**hGAPDH**	Forward	CGAGATCCCTCCAAAATCAA
	Reverse	CCTTCTCCATGGTGGTGAA

Table S2. Sequence of mimics for transfection

mimic	strand	Sequence (5’ – 3’)
**miR167**	Forward	ugaagcugccagcaugaucugc
	Reverse	gcagaucaugcuggcagcuuca

Table S3. Result of sRNA-seq for Cm-callus EVs

No.	Candidate ID	Sequence	ReadCount	RPTM	target	evalue	bitscore	miRNA family
1	Cma_e5567	UCGGACCAGGCUUCAUUCCCC	895	1869	cas-miR166c-3p	1.6.E-07	42.1	miR166
2	Cma_e3390	AAGCUCAGGAGGGAUAGCGCC	1070	2235	ath-miR390a-5p	1.6.E-07	42.1	miR390
3	Cma_e4039	UUAGAUUCACGCACAAACUCG	81	169	cas-miR403	1.6.E-07	42.1	miR403
4	Cma_e20377	UGAAGCUGCCAGCAUGAUCUG	59	123	tae-miR167c-5p	1.6.E-07	42.1	miR167
5	Cma_e15151	UGCACUGCCUCUUCCCUGGCUU	4006	8368	smo-miR408	1.8.E-07	42.1	miR408
6	Cma_e28173	UCGCUUGGUGCAGGUCGGGACC	56	117	nta-miR168a	1.8.E-07	42.1	miR168
7	Cma_e6855	UUCCACGGCUUUCUUGAACU	326	681	csi-miR396e-5p	5.8.E-07	40.1	miR396
8	Cma_e23865	GGAAUGUUGUCUGGCUCGAG	86	180	osa-miR166d-5p	5.8.E-07	40.1	miR166
9	Cma_e8569	GCAGUUCAAGAAAGCUGUGGAA	72	150	zma-miR396g-3p	2.7.E-06	38.2	Novel
10	Cma_e18746	CCUCAUUCCAUACAUCGUCCAA	56	117	gma-miR1507b	1.1.E-05	36.2	miR1507
11	Cma_e8070	AGGGGCGACCUGAGAACACA	439	917	zma-miR398a-5p	5.6.E-04	30.2	Novel
12	Cma_e23183	GAAACUCAGGAUGGAUAGCG	174	363	pta-miR390	2.0.E-03	28.2	Novel
13	Cma_e23277	GCAUGAUGAUGAUGAACUUA	32	67	ath-miR5658	2.0.E-03	28.2	Novel
14	Cma_e4476	AGCCAGGGGAGAGGCAGUGCA	17	36	bra-miR408-5p	2.0.E-03	28.2	Novel
15	Cma_e11255	GAUACGCUACAUCACUGUUCUC	21	44	fve-miR11300	3.0.E-03	28.2	Novel
16	Cma_e24428	GCUAGCGCUUGACUAAUAGAAU	18	38	pgi-miR6135e.2-5p	3.0.E-03	28.2	Novel
17	Cma_e16328	UUAGAUCUUUAUAGAUUUCGU	23	48	aly-miR4243	1.0.E-02	26.3	Novel
18	Cma_e3022	UAAAUAUUGACUUAGAAGAGA	23	48	bdi-miR5185l-3p	1.0.E-02	26.3	Novel
19	Cma_e22001	GGAGAUUUUGAAGGUAAAGGGC	20	42	tae-miR10518	1.0.E-02	26.3	Novel
20	Cma_e6891	ACAUCUACACUGCACUCAAUC	29	61	gma-miR397b-3p	3.8.E-02	24.3	Novel
21	Cma_e15990	AAGAAGAUCUUUGACAAAGUC	23	48	pab-miR11574	3.8.E-02	24.3	Novel
22	Cma_e24672	UAUUUUUUGAUUUUGGCAGGA	21	44	seu-miR11030a	3.8.E-02	24.3	Novel
23	Cma_e24750	UCAAUUUUUUCCUGUUCGAUU	19	40	gra-miR8722	3.8.E-02	24.3	Novel
24	Cma_e4819	UGUGCUCACUCUCUUCUGUCAA	180	376	aly-miR156d-3p	4.1.E-02	24.3	Novel
25	Cma_e2434	UGAUGAGCCAUCUUGAACAACU	57	119	mtr-miR5554a-5p	4.1.E-02	24.3	Novel
26	Cma_e16893	UUGCAGAACCAGGAAAACAUGU	35	73	aly-miR3448-5p	4.1.E-02	24.3	Novel
27	Cma_e10409	CUCCACAACUCUGAAGACCAUG	25	52	gma-miR9763	4.1.E-02	24.3	Novel
28	Cma_e20165	AAUUCUUUGUCUUUUCUGCCUG	22	46	pab-miR11520	4.1.E-02	24.3	Novel
29	Cma_e15189	UAGAGUCUUACAGUAGCCAAGG	22	46	pab-miR11581	4.1.E-02	24.3	Novel
30	Cma_e24899	CGGAGAGAAAAUAGUAGCCGGA	21	44	pab-miR1312a	4.1.E-02	24.3	Novel
31	Cma_e6579	GUUCUUUUAAUUGUUAGAGGAC	20	42	gra-miR8787	4.1.E-02	24.3	Novel
32	Cma_e27507	AUCAACUACACUCAAUCGGAUU	20	42	stu-miR397-3p	4.1.E-02	24.3	Novel
33	Cma_e4400	GUCAGGAUGGCUGGCCGAGU	40	84	gma-miR169o	1.4.E-01	22.3	Novel
34	Cma_e12106	AAGGACAUACUUCUACGGUGA	36	75	mtr-miR2591	1.5.E-01	22.3	Novel
35	Cma_e5791	UCUGAAAAUUCUGAUAUAUCC	27	56	gma-miR1512a-5p	1.5.E-01	22.3	Novel
36	Cma_e3043	CCAGCAAAUCAUCUCACACUU	27	56	bdi-miR7757-5p.2	1.5.E-01	22.3	Novel
37	Cma_e5219	GUGACUGUGACAUGUGAGCAG	27	56	gma-miR4372a	1.5.E-01	22.3	Novel

38	Cma_e20376	UUUGGAUUCAACUAACGCACC	23	48	mtr-miR5746	1.5.E-01	22.3	Novel
39	Cma_e3183	AAAUCGCCAGACAUCUUGUUU	23	48	aof-miR12160	1.5.E-01	22.3	Novel
40	Cma_e16311	UACCAAUACUACCAAAACUCC	22	46	ath-miR5638a	1.5.E-01	22.3	Novel
41	Cma_e9445	AGUAUAAUGCAGAUGCUCGAC	18	38	mtr-miR5554a-3p	1.5.E-01	22.3	Novel
42	Cma_e17810	AUAAACAGACGUCAUAUUGAA	21	44	gma-miR4362	1.5.E-01	22.3	Novel
43	Cma_e4720	UACAAUCAUUCAUUCUCAGUA	21	44	gma-miR5379	1.5.E-01	22.3	Novel
44	Cma_e20197	UGAGUUCAAGUGAAAAAGGAU	20	42	ppt-miR1051-5p	1.5.E-01	22.3	Novel
45	Cma_e17879	GAGGAACAAGGAGAAUGGAGCA	26	54	pab-miR11427a	1.6.E-01	22.3	Novel
46	Cma_e15188	UCCUGACAGCAUGUUUCUUCGA	23	48	tae-miR167b	1.6.E-01	22.3	Novel
47	Cma_e22117	AGAGGAAUUCAAGAAGGGGAUU	25	52	mtr-miR396c	1.6.E-01	22.3	Novel
48	Cma_e6698	GAUCCAAUACCACGCAUUUCGC	25	52	ppe-miR482d-3p	1.6.E-01	22.3	Novel
49	Cma_e5901	CGGUGGAGGAACGUCGCGCG	247	516	bdi-miR7732-3p	5.4.E-01	20.3	Novel
50	Cma_e3027	UGGUUGUUCCUGAUAAUUUA	22	46	osa-miR2095-5p	5.4.E-01	20.3	Novel
51	Cma_e8887	UACGCUUCCAUUCAUUCCCU	15	31	crt-miR166b	5.4.E-01	20.3	Novel
52	Cma_e4011	UGCGCGUCGUCUGGGCGAGGA	56	117	pab-miR11441a	5.9.E-01	20.3	Novel
53	Cma_e14811	UGCUCGCUCUCUUCUGUCUCC	62	130	ptc-miR7817b	5.9.E-01	20.3	Novel
54	Cma_e18905	GAUGUGUUCUCAUGACGCUCC	76	159	stu-miR398b-3p	5.9.E-01	20.3	Novel
55	Cma_e20455	AGACUACAUUUCGAACGGCAA	27	56	mtr-miR2679a	5.9.E-01	20.3	Novel
56	Cma_e8420	AGGACAGAAUCCUAUAAGGGG	24	50	ahy-miR3514-3p	5.9.E-01	20.3	Novel
57	Cma_e10377	GUUAGGUUCCAUCAUGCUGCU	23	48	ata-miR167f-3p	5.9.E-01	20.3	Novel
58	Cma_e9932	CCCUUUUGAGAUCGAUGCCGA	23	48	osa-miR5160	5.9.E-01	20.3	Novel
59	Cma_e20714	CAUUUUUCUGAAAAUUUGGUC	22	46	stu-miR8001b-5p	5.9.E-01	20.3	Novel
60	Cma_e6499	GAGUGACUUUGGGGCUUCGAC	21	44	cpa-miR477	5.9.E-01	20.3	Novel
61	Cma_e15984	GGUAUUCCUAGAGUUUGUGAG	21	44	sly-miR10535b	5.9.E-01	20.3	Novel
62	Cma_e20580	CACUUAACUUUGACAUCCCAA	21	44	gra-miR8762d	5.9.E-01	20.3	Novel
63	Cma_e21907	AGCCAGAAGGAGAGGGGAAAG	21	44	hvu-miR6192	5.9.E-01	20.3	Novel
64	Cma_e21986	UUCUCUUUUCCUCUCACCCAG	21	44	hbr-miR6169	5.9.E-01	20.3	Novel
65	Cma_e11169	GGGUAAUCAGAUCAUUUACUU	20	42	eun-miR167c-3p	5.9.E-01	20.3	Novel
66	Cma_e20597	AUUUCAAAGACAGCCACUCUA	20	42	lja-miR11128-3p	5.9.E-01	20.3	Novel
67	Cma_e2522	GGGUUCAUAUUUUCAGUCAAA	20	42	ath-miR774b-3p	5.9.E-01	20.3	Novel
68	Cma_e21474	UGGGAUUGAAUAGGAUGGAGAC	83	173	pab-miR11470	6.4.E-01	20.3	Novel
69	Cma_e7196	GGACGGACUGGGAGCGGCUCCU	10	21	zma-miR398b-5p	6.4.E-01	20.3	Novel
70	Cma_e20040	GUAGAGGCAGAUCCAAGUGAUA	40	84	pab-miR11487a	6.4.E-01	20.3	Novel
71	Cma_e26678	GAUUUUUGCAAACGAGUACUUU	27	56	aly-miR4238	6.4.E-01	20.3	Novel
72	Cma_e24434	AAGUAAUACACAAUCAUUCUCU	25	52	aly-miR4227	6.4.E-01	20.3	Novel
73	Cma_e9011	UACAAGUGAAGUAGUCAAAACU	25	52	stu-miR8017	6.4.E-01	20.3	Novel
74	Cma_e15152	UCUACGACCAUGAUAAUAACUU	23	48	stu-miR7982a	6.4.E-01	20.3	Novel
75	Cma_e9536	AAUCAGAAACUGACUUAUAAAU	23	48	osa-miR1861a	6.4.E-01	20.3	Novel
76	Cma_e7354	GGAGCAAAUUUGUCAACUGGUG	23	48	bra-miR9566-3p	6.4.E-01	20.3	Novel
77	Cma_e7090	ACGAAGAAACACUAUAUGAAUA	21	44	lja-miR11102-3p	6.4.E-01	20.3	Novel

78	Cma_e19889	UUCGAGUUUUGCUGACGAACGG	21	44	gra-miR8755	6.4.E-01	20.3	Novel
79	Cma_e28342	AUUUUUAGCAGAAUUUCAGAUC	21	44	gra-miR8718	6.4.E-01	20.3	Novel
80	Cma_e20634	CACCAGCUUCUGCACCAUUCUA	21	44	pab-miR11446	6.4.E-01	20.3	Novel
81	Cma_e14567	UAAUCAAAAAGUGGAACGCAUU	21	44	mtr-miR5284b	6.4.E-01	20.3	Novel
82	Cma_e16586	AAAAAUGGAAAUCCAGAGGGAU	11	23	pla-miR11604	6.4.E-01	20.3	Novel
83	Cma_e3011	UAACUUAUGAGAUCGACCGGGA	20	42	bdi-miR7784a-3p	6.4.E-01	20.3	Novel
84	Cma_e12772	GGUCCCCAAGAAUCAAUUUUAC	20	42	lja-miR11095-3p	6.4.E-01	20.3	Novel
85	Cma_e13087	AAGUUGCCAUCAUCAAGUCGAA	20	42	csi-miR172d-5p	6.4.E-01	20.3	Novel
86	Cma_e24960	AUUAGAGUAUGAGCUUGGCAGG	20	42	mes-miR399h	6.4.E-01	20.3	Novel
87	Cma_e14189	UAACGUCAAUGUUUCAACUAGU	19	40	stu-miR7980a	6.4.E-01	20.3	Novel
88	Cma_e17509	GAGUGCAAAGAGAGAGAUGGAG	20	42	csi-miR156f-5p	6.4.E-01	20.3	Novel
89	Cma_e28459	AUUUUAUUCUCUUCAUUCUACA	20	42	pab-miR11559	6.4.E-01	20.3	Novel
90	Cma_e25371	UCCGGGCAAGUUCUACUAUCUC	19	40	gma-miR169l-3p	6.4.E-01	20.3	Novel
91	Cma_e15584	CAAGGGUACUGCCAGCGGUCU	23	48	eun-miR167c-5p	2.3.E+00	18.3	Novel
92	Cma_e23165	CCUACACUUGAUGGCAUCGGU	22	46	pab-miR11559	2.3.E+00	18.3	Novel
93	Cma_e7782	GAGAGACACAGAGAGGAAGCG	21	44	pab-miR11477	2.3.E+00	18.3	Novel
94	Cma_e21663	CCUCUCACCAACUUCUGAUUU	17	36	bra-miR9561-5p	2.3.E+00	18.3	Novel
95	Cma_e25048	UACUCACUCACCCAUACUUGC	20	42	bna-miR6030	2.3.E+00	18.3	Novel
96	Cma_e18550	UAGCACGAGAUAGAUAAGAGA	23	48	csi-miR156i-5p	2.3.E+00	18.3	Novel
97	Cma_e16353	AGUUCAUUUAUGGAUCGAAAA	20	42	tae-miR5049-3p	2.3.E+00	18.3	Novel
98	Cma_e22875	GUUAUAUCUAAUUCUUGAGCU	19	40	osa-miR5145	2.3.E+00	18.3	Novel
99	Cma_e5220	CCAUGCUCCAUAUUACCUCCUU	24	50	gra-miR8778	2.5.E+00	18.3	Novel
100	Cma_e5334	AGUAUGCAUUUCCUUACUUAUU	23	48	bra-miR9558-3p	2.5.E+00	18.3	Novel
101	Cma_e9996	CGACUUGAGUCCGUACAUAUUC	21	44	aly-miR319a-5p	2.5.E+00	18.3	Novel
102	Cma_e16558	AAGCCCUUAUAGAGUCAAUGAA	23	48	bra-miR5722	2.5.E+00	18.3	Novel
103	Cma_e2743	AUUAUCCUUAAAUAAUUACCAG	21	44	lja-miR11108n-5p	2.5.E+00	18.3	Novel
104	Cma_e16636	GAGAUUGUGCAAAUAGGAGAGU	22	46	rgl-miR7799	2.5.E+00	18.3	Novel
105	Cma_e12925	CUCUCAAACGAUACUGAAGGCU	22	46	gra-miR8727	2.5.E+00	18.3	Novel
106	Cma_e23549	UCCCCGAUCCGAACGGGGGAUC	21	44	tae-miR1135	2.5.E+00	18.3	Novel
107	Cma_e23904	UCUAAGUCCAGAGGAAAUAACU	21	44	lja-miR11072b-5p	2.5.E+00	18.3	Novel
108	Cma_e2492	CUCUUCCUAGCUAUCUUCUUCC	21	44	bdi-miR7753-5p	2.5.E+00	18.3	Novel
109	Cma_e24570	CGUCGAAGGUCUAUGGUUCUGG	21	44	eun-miR159-5p	2.5.E+00	18.3	Novel
110	Cma_e16167	ACCAGUGAGAGACUGCUCCUUG	21	44	lja-miR7524	2.5.E+00	18.3	Novel
111	Cma_e23648	CCAAUCAUCCUAAAACUCCUCU	21	44	sbi-miR6220-5p	2.5.E+00	18.3	Novel
112	Cma_e11875	UUCAAUUAUUCUGACCUAUAUU	20	42	lja-miR11129-3p	2.5.E+00	18.3	Novel
113	Cma_e24493	UGCCUACGUACCUCUAUUUAUA	20	42	pab-miR11528	2.5.E+00	18.3	Novel
114	Cma_e17909	CAUCUUAGAAGGAGACAUAUCA	20	42	gma-miR10415	2.5.E+00	18.3	Novel

Table S4. Putative Human Target Genes of tae-miR167c-5p (from. psRNATarget V2)

Rank	Target Accession	Expectation	UPE	mRNA Target Aligned Fragment (5'-3')	Inhibition
1	NM_147180|PPP3R2	3	-1	UUAUGUCAUGUUGGUAGCUUUA	Cleavage
2	NM_001122853|MYOZ3	3	-1	GAUGAUGAUGAUGGCAGCUUUA	Cleavage
3	NM_006830|UQCR11	3.5	-1	GUUGAUCAUGCUGGUGGCUUGG	Cleavage
4	NM_012479|YWHAG	3.5	-1	AUGGAUCGUGUUGGUAUUUUCA	Cleavage
5	NM_015026|MON2	3.5	-1	UAUGAUCAUGCAGUUAGCUUCA	Translation
6	NM_080391|PTP4A2	4	-1	UCAGAGAAUGCUGGUAGCUUAA	Cleavage
7	NM_022340|ZFYVE20	4	-1	CAGGAUCGUGCUGGUAGCACCA	Cleavage
8	NM_003672|CDC14A	4	-1	GGGAAUCAUGUUGACAGUUUUA	Cleavage
9	NM_006004|UQCRH	4	-1	UUGGCUUAGGCUGGUAGCUUCU	Cleavage
10	NM_001089591|UQCRHL	4	-1	UUGGCUUAGGCUGGUAGCUUCU	Cleavage
11	NM_001109763|GSG1L	4	-1	GGGGACCAUGCGGGCAGAUUCA	Translation
12	NM_020880|ZNF530	4	-1	CUGAGUUCUGCUGGCAGCUUCC	Cleavage
13	NM_001122842|NCOA7	4	-1	UAACAGCAUACUGGCAGUUUCA	Cleavage
14	NM_001707|BCL7B	4	-1	ACGGAGC-UGCUGGCAGCUUCU	Cleavage
15	NM_022755|IPPK	4	-1	UGUUAUUAAGCUGACAGCUUCA	Cleavage
16	NM_001032731|OAS2	4.5	-1	GCAGAUCAUGCUUGCAGGUUUU	Translation
17	NM_004473|FOXE1	4.5	-1	GCAGGACGUGCUGGUAAUUUCA	Cleavage
18	NM_031961|KRTAP9-2	4.5	-1	GUGGAUCAUG-UGCCAGCUUCA	Cleavage
19	NM_199296|ISM2	4.5	-1	CCAUGUCAUGCUGCCAGCAUCA	Cleavage
20	NM_182509|ISM2	4.5	-1	CCAUGUCAUGCUGCCAGCAUCA	Cleavage
21	NM_001200001|NOTCH2	4.5	-1	GUGGUUUAUGUUGUCAGUUUUA	Cleavage
22	NM_001098504|DDX17	4.5	-1	UCAGGUUCUGCUGUUGGCUUCA	Cleavage
23	NM_032329|ING5	4.5	-1	GUGGGUCACUGUGGCAGCUUCA	Cleavage
24	NM_001963|EGF	4.5	-1	CAAGGUCGUGCUGGUAAUUUUG	Cleavage
25	NM_001099455|CPPED1	4.5	-1	UUGGAUCAUGGAGGCAGUUUCU	Translation
26	NM_001080533|UNC119B	4.5	-1	AUGCCACAUGCUGGCAGCUUUC	Cleavage
27	NM_001080509|TSPAN11	4.5	-1	AGUAUCCAUGCUGGCAGCUUUU	Cleavage
28	NM_001199805|KLRC4-KLRK1	4.5	-1	GGGGGUCAUGCUGCCACUUUUA	Cleavage
29	NM_152440|C12orf66	4.5	-1	GAAGAUCCUCAUGGCAGCUUUA	Cleavage
30	NM_001142505|ABCG4	4.5	-1	GUGCUCCAUGCUGGCAUCUUCA	Cleavage
31	NM_001253697|ERBB2IP	4.5	-1	GAAGGUGGUGCUGGUGGGUUCA	Cleavage
32	NM_173551|ANKS6	4.5	-1	CUACUAUGUGCUGGCAGCUUUA	Cleavage
33	NM_001206840|TGOLN2	4.5	-1	UUUAUUUAUGCUGGUGGCUUCC	Cleavage
34	NM_001206841|TGOLN2	4.5	-1	UUUAUUUAUGCUGGUGGCUUCC	Cleavage
35	NM_006464|TGOLN2	4.5	-1	UUUAUUUAUGCUGGUGGCUUCC	Cleavage
36	NM_199452|ZNF365	4.5	-1	CUACAACAUGCUGGCAACUUCC	Cleavage
37	NM_001166412|SMOC2	4.5	-1	UGGAAUCAUGUUGGAAGCUCCA	Cleavage

38	NM_001195283|FLVCR2	4.5	-1	UGUGAUCAUGCUGGGGGCUACC	Cleavage
39	NM_001082577|RBFOX2	4.5	-1	AUUGAUCAUGUUGCUGGCUUUU	Cleavage
40	NM_001031695|RBFOX2	4.5	-1	AUUGAUCAUGUUGCUGGCUUUU	Cleavage
41	NM_001166163|PPP1R9A	4.5	-1	CUGGAUCACACUGGAAGUUUCA	Cleavage
42	NM_001164440|ANKRD33B	4.5	-1	AGCGGUCAUGCUGGGAGCUGCC	Cleavage
43	NM_018383|WDR33	4.5	-1	GCCGCUGGUGCUGGAAGUUUCA	Cleavage
44	NM_018383|WDR33	4.5	-1	UUUCAUUACAUUGGCAGCUUCA	Cleavage
45	NM_001031672|CYB5RL	4.5	-1	GGGGGUGGUGCUGGAGGUUUCA	Cleavage
46	NM_018958|NPAP1	4.5	-1	UAUGGUCAUGCUUCUAGUUUCA	Translation
47	NM_181644|MFSD4	4.5	-1	UGAUAGCAUGUUGGUAGCUUGA	Cleavage
48	NM_004521|KIF5B	4.5	-1	UAGGAUCUUGCUGGCAGAAUUA	Cleavage
49	NM_001166252|LPPR4	4.5	-1	UAAGAAGAUGCUGGCUGCUUUG	Cleavage
50	NM_007192|SUPT16H	5	-1	GCAGAUUGGGCUGGUGCCUUCG	Cleavage
51	NM_004093|EFNB2	5	-1	GCAGGUCACACUGGGGGCUUCA	Cleavage
52	NM_000949|PRLR	5	-1	CACUAUCAUGCUGGCAGCUGUU	Cleavage
53	NM_002972|SBF1	5	-1	GCGGAUCGAGCAGGAGGCUUCA	Translation
54	NM_033191|KRTAP9-4	5	-1	GUGGAUCAUG-UGCCAGCUUCG	Cleavage
55	NM_031962|KRTAP9-3	5	-1	GUGGAUCAUG-UGCCAGCUUCG	Cleavage
56	NM_001114089|ENTPD6	5	-1	ACAGGCCGUGCUGGCACUUUCU	Cleavage
57	NM_182557|BCL9L	5	-1	CCAGAU-GUGCUGGCAGCUUAG	Cleavage
58	NM_018482|ASAP1	5	-1	CUGGGUUGUGACUGGCAGCUUUA	Cleavage
59	NM_014951|ZNF365	5	-1	ACAGACCAUGCUGCCAGCCUCU	Cleavage
60	NM_197975|BTNL3	5	-1	GUGAAUCAUGCUUGCAGGUUUG	Translation
61	NM_001198844|RBM4	5	-1	GUAUUUCAUCCUGGCAGCCUCA	Cleavage
62	NM_002137|HNRNPA2B1	5	-1	GUAGUUCUUGUUGGUGCCUUCA	Cleavage
63	NM_001039656|MTL5	5	-1	GUGGGCCAUGCUGCUUGCUUCA	Cleavage
64	NM_145200|CABP4	5	-1	GUGGCUCAUGCUUGUAGUUCCA	Translation
65	NM_175709|CBX7	5	-1	UUGGAACAUGGCUGGCAGCUUCC	Cleavage
66	NM_194320|ZNF169	5	-1	UCAGUUCAAGCUGGCAGCAUUU	Cleavage
67	NM_032818|ARHGEF39	5	-1	GCGGG-CAUGCUGGUGGCCUCA	Cleavage
68	NM_017691|LRRC49	5	-1	ACAGUUGAUUUUGGCAGUUUUA	Cleavage
69	NM_001190956|ADAM18	5	-1	AUAGAAUUCUUGCUGGCAGUUUCU	Cleavage
70	NM_138492|PRELID2	5	-1	GGAUACAGUGUUGGCAGCUUCA	Cleavage
71	NM_001039673|YIF1B	5	-1	GUGGUUGGUGCAGGCAGCUUCU	Translation
72	NM_001145463|YIF1B	5	-1	GUGGUUGGUGCAGGCAGCUUCU	Translation
73	NM_024012|HTR5A	5	-1	CCACAUUUUUCUGGUGGCUUCA	Cleavage
74	NM_022748|TNS3	5	-1	UCAGGAGGUGCUGGCAUUUUCA	Cleavage
75	NM_152667|NANP	5	-1	ACAGAGGAUGUUUGUAGUUUCA	Translation
76	NM_138572|TAF8	5	-1	GCUGGGCAUGCUGAUAGCUUUU	Cleavage
77	NM_006544|EXOC5	5	-1	UUAGGACAUUAUGGCAGCUUUA	Cleavage

78	NM_203412|UBL4B	5	-1	CCCUCCUGUGCUGGCAGCUUUG	Cleavage
79	NM_173664|ARL10	5	-1	CCUUCUCAUGUGGGCAGCUUCU	Translation
80	NM_014238|KSR1	5	-1	ACUGAGCAUGCUGGGAGCUUGG	Cleavage
81	NM_001098812|SEPT8	5	-1	CAGGAUCAUGUUGGCAUUUACU	Cleavage
82	NM_001098813|SEPT8	5	-1	CAGGAUCAUGUUGGCAUUUACU	Cleavage
83	NM_019118|TMEM234	5	-1	GACUAUCAUGGUGGCAGGUUCC	Cleavage
84	NM_014553|TFCP2L1	5	-1	GUGGAUGAAGCUGGUGGCCUUA	Cleavage
85	NM_001039547|GK5	5	-1	CAAAAUCGUGUUGGUGGUUUUU	Cleavage
86	NM_153442|GPR26	5	-1	CAGGGUAGUGGUGGCUGCUUCA	Cleavage
87	NM_006330|LYPLA1	5	-1	AUAGAUGAAGCAGGUAGCUUCU	Translation
88	NM_006768|BRAP	5	-1	UGAGAGUGUGCUGGGACCUUCA	Cleavage
89	NM_013272|SLCO3A1	5	-1	AUUUAUUAUGCUGGUUGCUUUU	Cleavage
90	NM_014997|KLHDC10	5	-1	GUUCUUCAUUCUGGCAGUUUUG	Cleavage
91	NM_002585|PBX1	5	-1	CGUUUUCAUGCUGGUGGUUUGA	Cleavage
92	NM_001204961|PBX1	5	-1	CGUUUUCAUGCUGGUGGUUUGA	Cleavage
93	NM_017628|TET2	5	-1	ACUGAUCAUGGUGGUAGUUGCU	Cleavage
94	NM_133374|ZNF618	5	-1	CACCAUCCUGCUUGGCAGCUUCA	Translation
95	NM_001261436|CDK15	5	-1	GAUAAUUAUGCUGUCAGCCUCG	Cleavage
96	NM_002883|RANGAP1	5	-1	UAAAGUCGUG-UGGCAGCUUCA	Cleavage

Table S5. Putative Human Target Genes of tae-miR167c-5p (from. psRNATarget V1)

Rank	Target Accession	Expectation	UPE	mRNA Target Aligned Fragment (5'-3')	Inhibition
1	NM_031961|KRTAP9-2	2.5	11.3	GUGGAUCAUG-UGCCAGCUUCA	Translation
2	NM_147180|PPP3R2	3	18.6	UUAUGUCAUGUUGGUAGCUUUA	Cleavage
3	NM_033191|KRTAP9-4	3	14.3	GUGGAUCAUG-UGCCAGCUUCG	Translation
4	NM_031962|KRTAP9-3	3	13.0	GUGGAUCAUG-UGCCAGCUUCG	Translation
5	NM_020731|AHRR	3	22.0	AGUGAUCAUGGCUGGACAGCUUCA	Cleavage
